# Effect of Brassinolide on the Growth and Physiological Indicators of Foxtail Millet Under Cyhalofop-Butyl Damage

**DOI:** 10.3390/plants14223421

**Published:** 2025-11-08

**Authors:** Chunyan Hu, Jiaxin Dong, Jingtao Yuan, Suqi Shang, Xutao Zhai, Yinyuan Wen, Xi’e Song, Juan Zhao, Hui Cao, Shuqi Dong

**Affiliations:** 1 College of Plant Protection, Shanxi Agricultural University, Jinzhong 030800, China; hucy4216@sxau.edu.cn (C.H.); yuanjingtao2025@163.com (J.Y.); xutaozhai@163.com (X.Z.); 2 College of Agriculture, Shanxi Agricultural University, Jinzhong 030800, China; djx199907@163.com (J.D.); ssq2025@163.com (S.S.); wenyinyuan@126.com (Y.W.); sxndsxe@163.com (X.S.); sxndzhaojuan@163.com (J.Z.); 3 Jinzhong Agricultural High-Tech Zone Seed Industry Development Co., Ltd., Jinzhong 030800, China; 4 Special Orphan Crops Research Center of the Loess Plateau, MARA, Shanxi Agricultural University, Jinzhong, 030800, China

**Keywords:** foxtail millet, brassinolide, cyhalofop-butyl, alleviate drug damage

## Abstract

Cyhalofop-butyl is a gramineous herbicide with good control effect, but it causes some damage when used in foxtail millet fields. Brassinolide (BR) is a type of plant growth hormone that can enhance the stress resistance of crops and plays a crucial role in eliminating and alleviating herbicide damage. To investigate the alleviating effect of BR on cyhalofop-butyl damage in foxtail millet, a study was conducted using Jingu 21 as the test material, combining pot experiments and field experiments. All test treatments were sprayed with cyhalofop-butyl at a concentration of 67.5 g a.i./ha. Three BR spraying times were set: the same day as cyhalofop-butyl spraying (D1), one day later (D2), and three days later (D3). Four BR concentrations were set—0 mg/L (C0), 0.05 mg/L (C1), 0.1 mg/L (C2), and 0.2 mg/L (C3)—resulting in a total of 12 treatments. The results showed that after BR spraying, all agronomic trait indicators of Jingu 21 in both pot and field experiments were alleviated. Compared with the control treatment, the activities of superoxide dismutase (SOD), peroxidase (POD), and catalase (CAT) increased to varying degrees, the malondialdehyde (MDA) content decreased, and the drug damage level was alleviated to different extents. In addition, spraying BR can increase the yield of Jingu 21 under cyhalofop-butyl herbicide damage. The results of all indicators indicated that spraying BR one day after cyhalofop-butyl spraying had the best effect. Therefore, spraying BR at a concentration of 0.1 mg/L can effectively alleviate the damage of Jingu 21 plants. It is recommended that when using BR to alleviate damage in foxtail millet, the application should be spaced one day apart from the herbicide spraying.

## 1. Introduction

Foxtail millet originated from the Yellow River Basin and has characteristics such as a short growth period, drought resistance, and barren tolerance. Its planting area in northern China is second only to that of wheat and corn, and it is also a characteristic crop in Shanxi Province. During the growth process, there is a competitive relationship between weeds and foxtail millet. The competition for growth resources seriously affects the growth, development, yield, and quality of foxtail millet [1], restricting its production. However, there are currently few types of herbicides that can be safely applied to foxtail millet fields. Additionally, due to the influence of factors such as the environment, equipment, soil, and human factors, the improper application of herbicides can cause varying degrees of damage to crops.

Cyhalofop-butyl is an acetyl-CoA carboxylase inhibitor. After foliar spraying, it is absorbed by the leaves and leaf sheaths of plants and transported through the phloem to accumulate in the apical meristem of the stems, leading to the destruction of the membrane system [2]. When cyhalofop-butyl within a certain concentration range is used in foxtail millet fields, foxtail millet will suffer from damage, showing symptoms such as stunted plants, yellowing leaves, the appearance of phytotoxic spots, and even failure to grow new leaves and the blackening of stems appear. A relatively high concentration can cause the death of foxtail millet plants. These are manifestations of damage, pathological changes, and senescence of plants [3].

Brassinolide belongs to steroid compounds and is a plant hormone with strong physiological activity [4]. It plays an important role in plant growth, development [5], stress response and has an important regulatory effect on plant growth and development [6]. The treatment of seeds by soaking in BR or spraying BR at the seedling stage can promote cell division and growth [7,8], enhance root development [9,10], improve the utilization rate of water and fertilizers, increase antioxidant capacity [11], strengthen photosynthesis [12,13], improve crop quality [14], and increase crop yield. At the same time, BR has a regulatory effect on the agronomic traits of plants [15,16]. A low concentration of BR promotes root growth, while a high concentration of BR can increase the number of primary roots but can shorten the root length and cause root curling [17].

Under external stress conditions, plant growth and development will be hindered, and the antioxidant system will be damaged. Spraying BR can increase the activity of relevant antioxidant enzymes, reduce the malondialdehyde (MDA) content, improve photochemical activity, increase the content of endogenous auxin in plants, enhance the cold resistance, drought resistance, and salt tolerance of crops, and alleviate the damage caused by herbicides to crops [18,19]. Studies have shown that BR can improve the growth parameters of wheat, help enhance the salt tolerance of wheat [20], alleviate the damage to plants during high- and low-temperature stress and the rewarming recovery process [21], improve the cold resistance of crops [22], and alleviate the reduction in the number of spikelets of rice caused by high temperatures [23]. When the content of metal ions in the soil is too high, BR can increase the efflux of heavy metals from crops, thereby reducing the absorption of heavy metals by plants [24,25]. When crops are subjected to drought stress, BR can increase the leaf water potential of plants, reduce the transpiration intensity of crop leaves, and affect the water and fertilizer absorption effect of plants [26].

There are many types of herbicides on the market, but the degree of damage caused by different types of herbicides varies when applied to different crops. There are relatively few studies on the application of BR in foxtail millet production and experiments, especially those focusing on alleviating the damage caused by herbicides on foxtail millet. Currently, there is no research on using BR to alleviate the damage caused by cyhalofop-butyl on foxtail millet. Foxtail millet is a major food crop grown in Shanxi Province. As a safe herbicide for rice fields, cyhalofop-butyl can control important gramineous weeds, but it causes a certain level of damage to foxtail millet when used in foxtail millet fields. Therefore, the focus of this experiment is to investigate the effects of different concentrations of BR and spraying times on the growth and physiological indicators of foxtail millet under cyhalofop-butyl damage.

## 2. Results

### 2.1. Effect of BR Spraying on Agronomic Traits of Jingu 21 Under Cyhalofop-Butyl Damage

**Figure 1:**
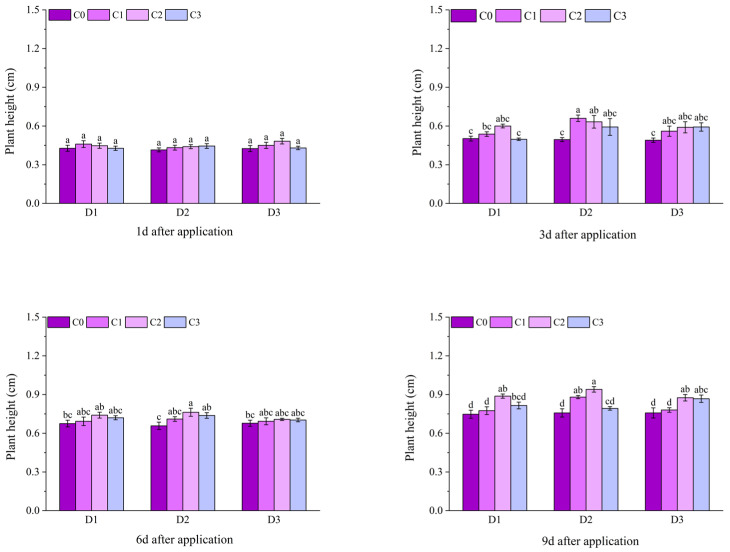

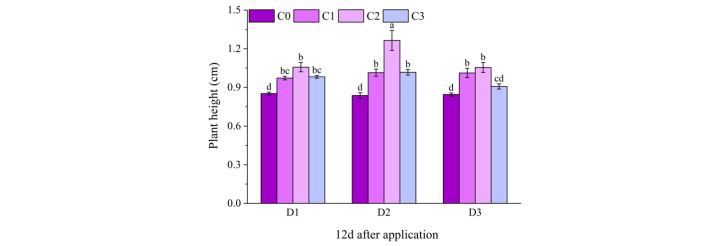
Effects of spraying BR on plant height of potted foxtail millet under cyhalofop-butyl damage. Comparison between treatments of different concentrations on the same day, with lowercase letters representing a significant difference (*p* < 0.05).

At 1 d after application, the difference in plant height of potted Jingu 21 among different treatments was small. At 3 d after application, compared with D1C0, the plant heights of the treatments with BR spraying under D1 increased by 7.76%, 15.87%, and 14.27%, respectively. Compared with D2C0, the plant heights of the treatments with BR spraying under D2 increased by 9.11%, 23.87%, and 7.50%, respectively. Starting from 6 d after application, the difference in plant height among different treatments gradually became significant. At 9 d after application, the plant heights of all treatments with BR spraying under D1 and D2 were significantly higher than that of the C0 treatment, and the plant height of the D3C2 treatment was significantly higher than that of the D3C0 treatment. At 12 d after application, the plant heights of all treatments with BR spraying were significantly higher than that of the C0 treatment. Compared with D1C0, the plant heights of the treatments with BR spraying under D1 increased by 31.19%, 48.37%, and 33.75%, respectively. Compared with D2C0, the plant heights of the treatments with BR spraying under D2 increased by 31.82%, 49.17%, and 33.48%, respectively. Among these, the D2C2 treatment had the highest value, reaching 33.75 cm. Compared with D3C0, the plant heights of the treatments with BR spraying under D3 increased by 22.88%, 36.19%, and 22.99%, respectively (Figure 1).

**Figure 2:**
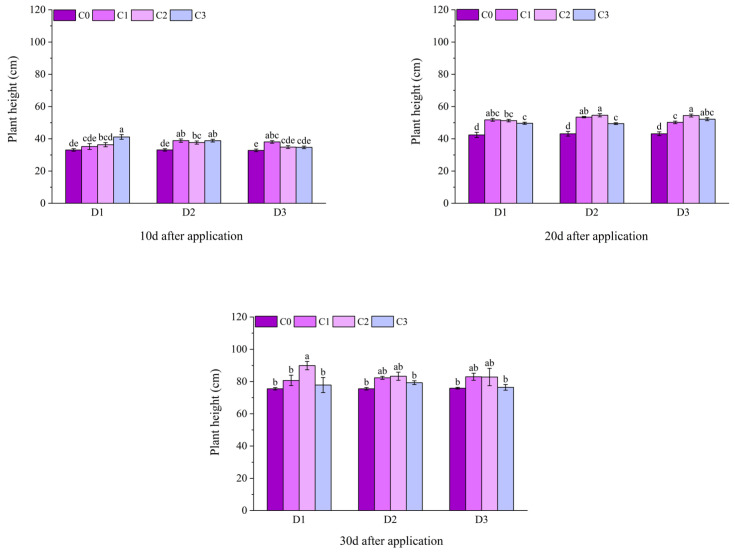
Effects of spraying BR on plant height of field foxtail millet under cyhalofop-butyl damage. Comparison between treatments of different concentrations on the same day, with lowercase letters representing a significant difference (*p* < 0.05).

At 10 d after the application of field Jingu 21, the plant heights of all treatments with BR spraying under D2 were significantly higher than those following D2C0 treatment, increasing by 17.36%, 13.74%, and 17.28%, respectively, in comparison. At 20 d after application, the plant heights of all treatments with BR spraying under D1, D2, and D3 were significantly different from those that underwent the C0 treatment. Compared with D1C0, the plant heights of the treatments with BR spraying under D1 increased by 21.98%, 20.86%, and 16.91%, respectively. Compared with D2C0, the plant heights of the treatments with BR spraying under D2 increased by 23.94%, 26.61%, and 14.55%, respectively. Compared with D3C0, the plant heights of the treatments with BR spraying under D3 increased by 16.35%, 26.20%, and 20.92%, respectively. At 30 d after application, the plant height of the D1C2 treatment was significantly higher than that of the D1C0 treatment, and there was no significant difference between other treatments with BR spraying and the C0 treatment (Figure 2).

**Figure 3:**
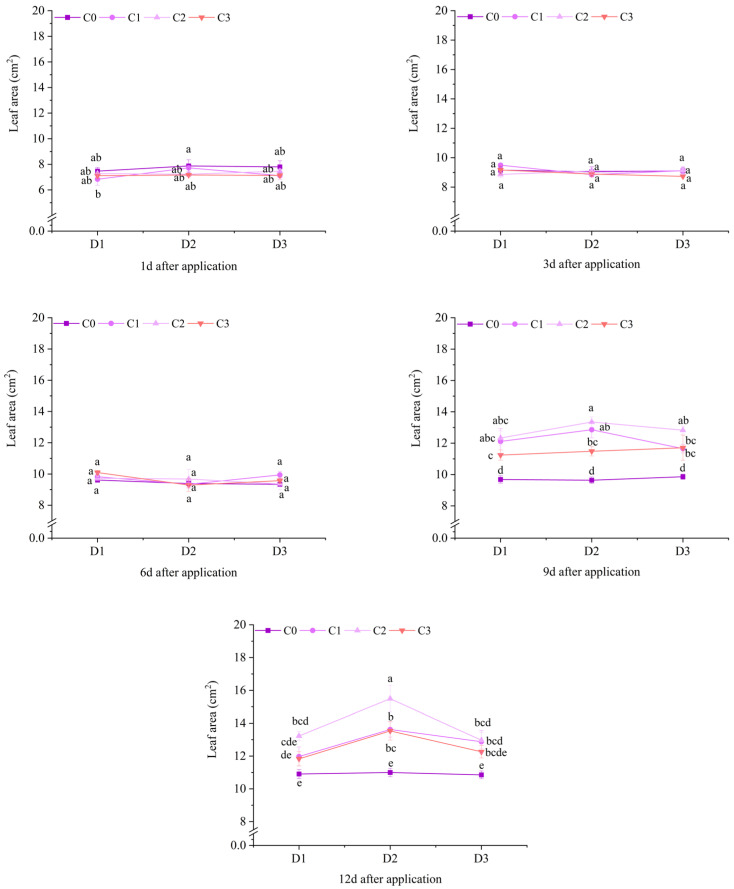
Effects of spraying BR on leaf area of potted foxtail millet under cyhalofop-butyl damage. Comparison between treatments of different concentrations on the same day, with lowercase letters representing a significant difference (*p* < 0.05).

At 1 d, 3 d, and 6 d after application, there was no significant difference in the leaf area of potted Jingu 21 among different treatments. Starting 9 d after application, the difference in leaf area among different treatments gradually became significant. At 9 d and 12 d after application, the leaf areas of the C1 and C2 treatments under D1 and D3, and all treatments with BR spraying under D2 were significantly higher than that of the C0 treatment. At 9 d after application, the leaf areas increased by 25.03%, 27.28%, 18.15%, 30.09%, 33.35%, 38.46% and 19.11%, respectively, compared with the C0 treatment. At 12 d after application, the leaf areas increased by 9.79%, 21.27%, 18.63%, 19.46%, 23.87%, 40.85%, and 23.07%, respectively, compared with the C0 treatment (Figure 3).

**Figure 4:**
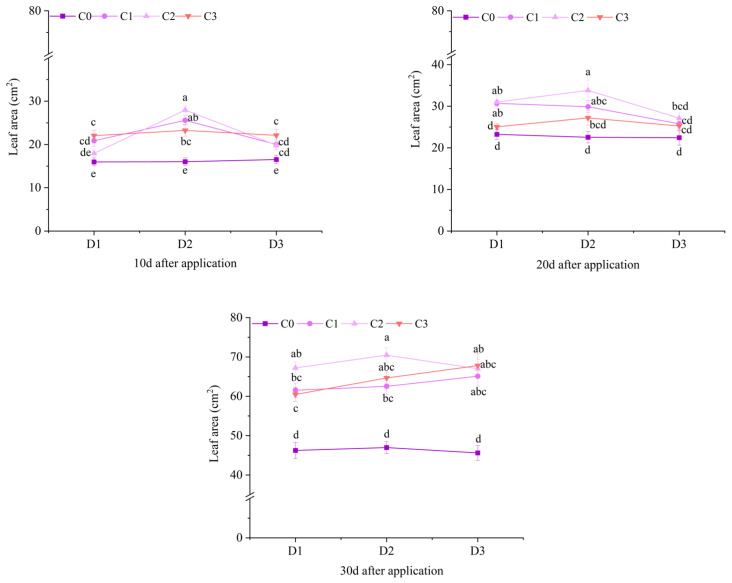
Effects of spraying BR on leaf area of field foxtail millet under cyhalofop-butyl damage. Comparison between treatments of different concentrations on the same day, with lowercase letters representing a significant difference (*p* < 0.05).

At 10 d after treatment of field Jingu 21, the leaf areas of all treatments with BR spraying under D2 and D3 were significantly different from that of the C0 treatment, among which the D2C2 treatment had the highest value, increasing by 74.47% compared with the D2C0 treatment. At 20 d after application, the leaf areas of the C1 and C2 treatments under D1 and D2 were significantly higher than that of the C0 treatment. At 30 d after application, the leaf areas of the treatments with BR spraying under D1 increased by 33.05%, 45.34% and 30.72%, respectively, compared with D1C0. Compared with D2C0, the leaf areas of the treatments with BR spraying under D2 increased by 33.10%, 49.99% and 37.65%, respectively. Compared with D3C0, the leaf areas of the treatments with BR spraying under D3 increased by 42.76%, 46.96% and 48.65%, respectively (Figure 4).

**Figure 5:**
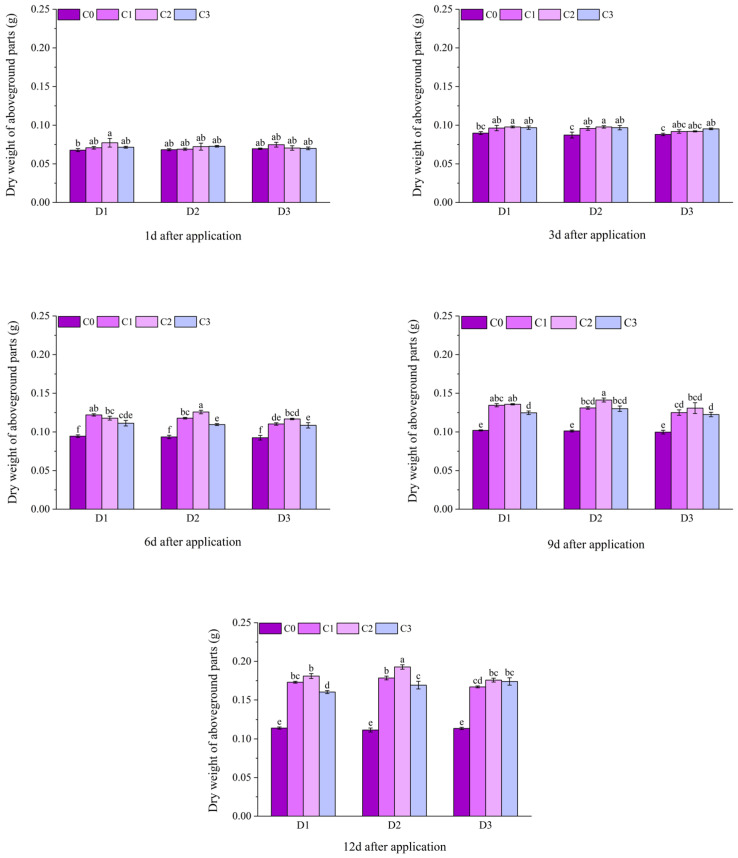
Effects of spraying BR on dry weight of aboveground parts of potted foxtail millet under cyhalofop-butyl damage. Comparison between treatments of different concentrations on the same day, with lowercase letters representing a significant difference (*p* < 0.05).

At 1 d after application, among the dry weight of aboveground parts of potted Jingu 21, only the D1C2 treatment was significantly higher than the D1C0 treatment, and there was no significant difference among other treatments. At 3 d after application, the dry weight of aboveground parts of the D1C2 and D3C3 treatments were significantly higher than that of the C0 treatment, and there were significant differences between all treatments under D2 and the C0 treatment. At 6 d, 9 d and 12 d after application, the dry weight of aboveground parts of all treatments with BR spraying was significantly higher than that of the C0 treatment, among which the D2C2 treatment had the highest value, increasing by 34.55%, 39.49% and 72.83%, respectively, compared with the D2C0 treatment (Figure 5).

**Figure 6:**
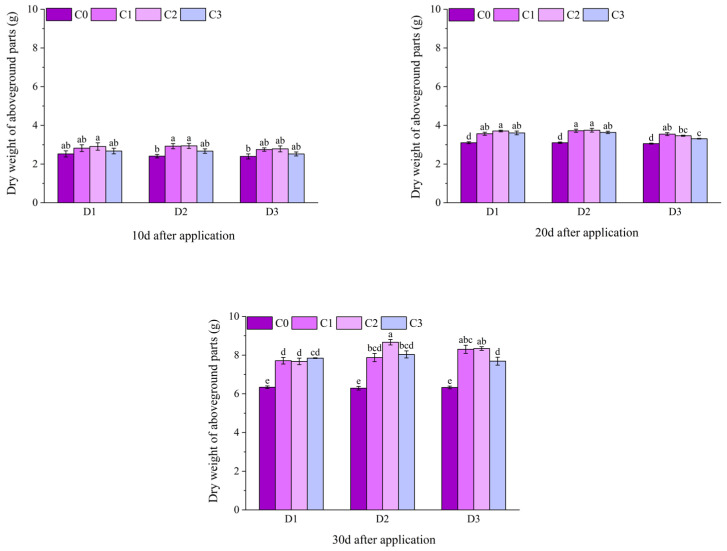
Effects of spraying BR on dry weight of aboveground parts of field foxtail millet under cyhalofop-butyl damage. Comparison between treatments of different concentrations on the same day, with lowercase letters representing a significant difference (*p* < 0.05).

At 10 d after the application of field Jingu 21, the dry weight of aboveground parts of the D2C1 and D2C2 treatments with significant differences, increased by 21.50% and 22.12%, respectively, compared with the D2C0 treatment. At 20 d after application, there were significant differences in the dry weight of aboveground parts between all treatments with BR spraying and the C0 treatment. Compared with D1C0, the dry weight of aboveground parts of the treatments with BR spraying under D1 increased by 14.91%, 19.50% and 16.28%, respectively. Compared with D2C0, the dry weight of aboveground parts of the treatments with BR spraying under D2 increased by 19.98%, 20.79% and 17.16%, respectively. Compared with D3C0, the dry weight of aboveground parts of the treatments with BR spraying under D3 increased by 16.03%, 13.25% and 8.26%, respectively. At 30 d after application, the dry weight of aboveground parts of the D2C2 treatment reached the maximum value of 8.67 g, increasing by 37.80% compared with the D2C0 treatment (Figure 6).

At 12 d after application, the BR spraying time had a significant effect on the plant height of potted Jingu 21, and an extremely significant effect on the leaf area and dry weight of aboveground parts. At 9 d and 12 d after application, the BR concentration had an extremely significant effect on the plant height, leaf area and dry weight of aboveground parts of potted Jingu 21. At 12 d after application, the interaction between BR spraying time and concentration had an extremely significant effect on the dry weight of aboveground parts (Table 1).

At 30 d after application, the BR spraying time had an extremely significant effect on the dry weight of aboveground parts of field Jingu 21. At 10 d, 20 d and 30 d after application, the BR concentration had an extremely significant effect on the plant height, leaf area, and dry weight of aboveground parts of Jingu 21. At 30 d after application, the interaction between BR spraying time and concentration had an extremely significant effect on the dry weight of aboveground parts of Jingu 21 (Table 2).

### 2.2. Effect of BR Spraying on Antioxidant Enzymes of Jingu 21 Under Cyhalofop-Butyl Damage

**Figure 7:**
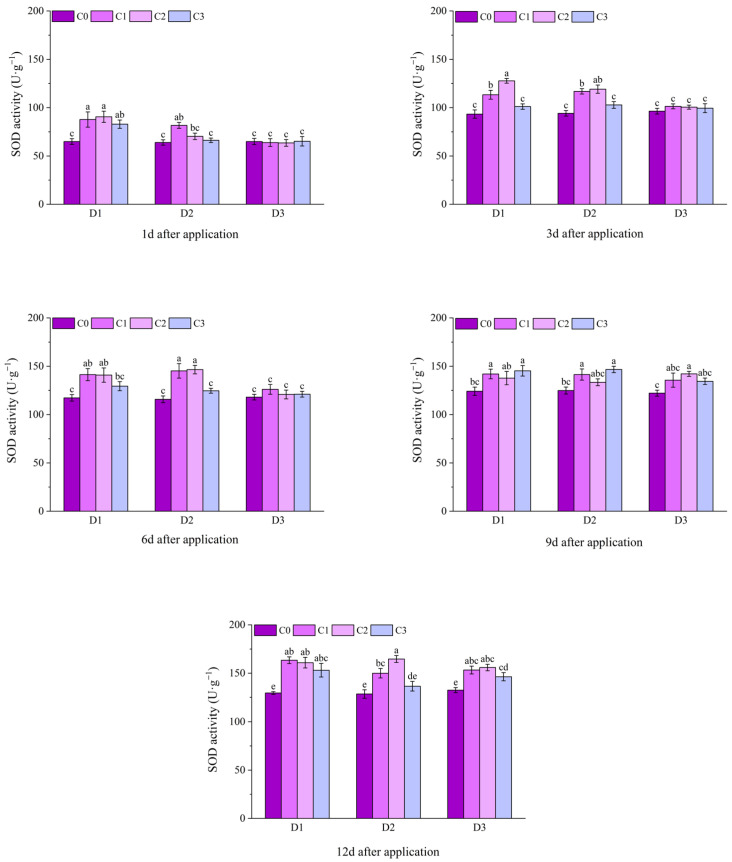
Effects of spraying BR on SOD activity of potted foxtail millet under cyhalofop-butyl damage. Comparison between treatments of different concentrations on the same day, with lowercase letters representing a significant difference (*p* < 0.05).

Within 1–12 d after application, the SOD activity of potted Jingu 21 showed a gradually increasing trend. At 1 d after application, the SOD activities of all treatments with BR spraying under D1 and the D2C1 treatment were significantly higher than that of the C0 treatment, and there was no significant difference between other treatments and the C0 treatment. At 3 d and 6 d after application, the SOD activities of the C1 and C2 treatments under D1 and D2 were significantly higher than that of the C0 treatment. At 3 d after application, the SOD activities increased by 21.37%, 36.82%, 24.15% and 26.59%, respectively, compared with the C0 treatment. At 6 d after application, the SOD activities increased by 20.15%, 10.32%, 25.46% and 26.56%, respectively, compared with the C0 treatment, and there was no significant difference between other treatments and the C0 treatment. At 9 d after application, the SOD activities of the C1 and C3 treatments under D1 and D2, and the C2 treatment under D3 were significantly higher than that of the C0 treatment, increasing by 14.42%, 17.10%, 13.27%, 17.46% and 16.42%, respectively, compared with the C0 treatment, and there was no significant difference between other treatments and the C0 treatment. At 12 d after treatment, except for the D2C3 treatment, the SOD activity of all other treatments with BR spraying were significantly higher than that of the C0 treatment (Figure 7).

**Figure 8:**
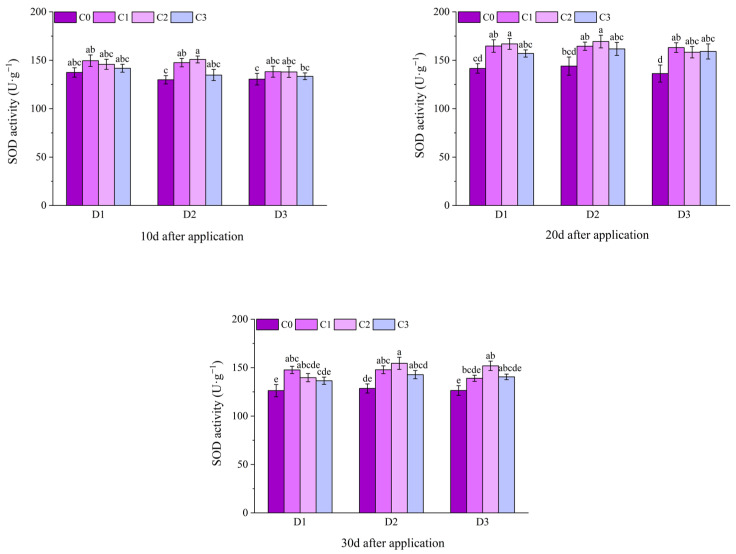
Effects of spraying BR on SOD activity of field foxtail millet under cyhalofop-butyl damage. Comparison between treatments of different concentrations on the same day, with lowercase letters representing a significant difference (*p* < 0.05).

At 10 d after application of field Jingu 21, the SOD activities of the C1 and C2 treatments under D2 were significantly higher than that of the D2C0 treatment. At 20 d and 30 d after application, the SOD activities of the C2 treatments under D1, D2, and D3 were significantly higher than that of the C0 treatment. At 30 d after application, the SOD activity of the D2C2 treatment reached the maximum, increasing by 20.22%, a significant difference compared with the D2C0 treatment (Figure 8).

**Figure 9:**
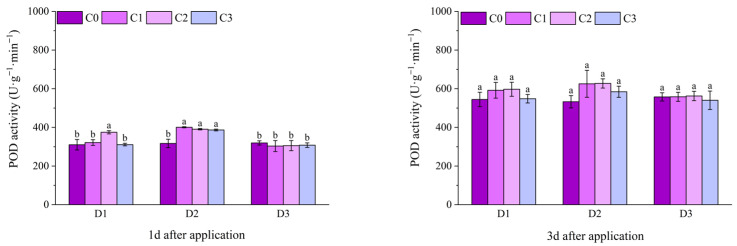

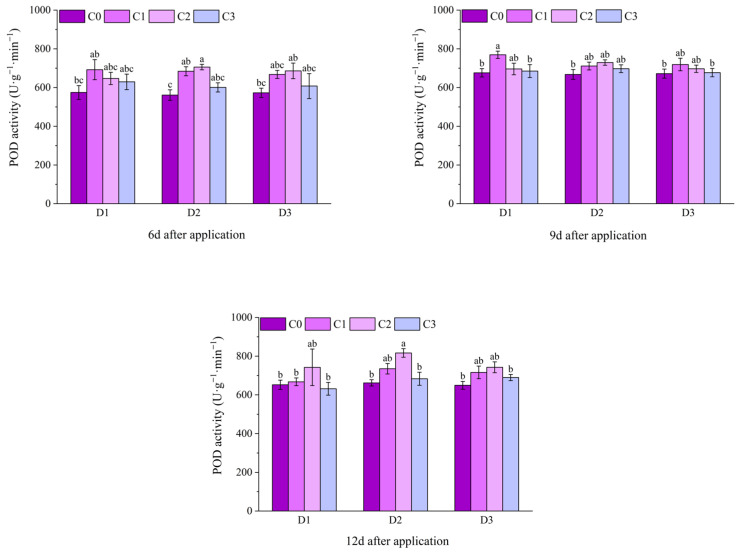
Effects of spraying BR on POD activity of potted foxtail millet under cyhalofop-butyl damage. Comparison between treatments of different concentrations on the same day, with lowercase letters representing a significant difference (*p* < 0.05).

At 1 d after application, the POD activities of the C2 treatments under D1 and D2 of potted Jingu 21 were significantly higher than that of the C0 treatment. At 3 d after application, there was no significant difference in POD activity among all treatments. At 6 d after application, except for the C1 and C2 treatments under D2, there was no significant difference in POD activity between other treatments and the C0 treatment, and the D2C2 treatment had the highest value, increasing by 25.81% compared with the D2C0 treatment. At 9 d after application, the POD activity of the D1C1 treatment increased by 13.78% compared with the D1C0 treatment, and there was no significant difference between other treatments and the C0 treatment. At 12 d after application, the POD activity of the D2C2 treatment was significantly higher than that of the D2C0 treatment and reached the maximum value, and there was no significant difference between other treatments and the C0 treatment (Figure 9).

**Figure 10:**
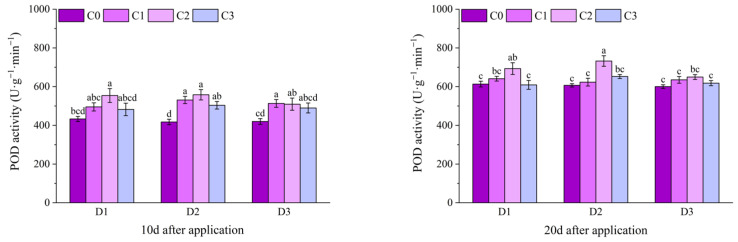

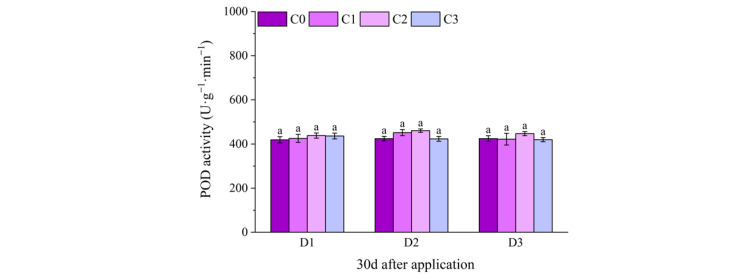
Effects of spraying BR on POD activity of field foxtail millet under cyhalofop-butyl damage. Comparison between treatments of different concentrations on the same day, with lowercase letters representing a significant difference (*p* < 0.05).

At 10 d and 20 d after application of field Jingu 21, the POD activity of the D1C2 treatment was significantly higher than that of the D1C0 treatment, increasing by 27.95% and 13.11%, respectively, in comparison. At 10 d after application, the POD activities of all treatments with BR spraying under D2, and the C1 and C2 treatments under D3 were significantly higher than that of the C0 treatment. At 30 d after application, there was no significant difference in POD activity among all treatments. With the passage of time, the POD activity of Jingu 21 showed a trend of first increasing and then decreasing (Figure 10).

**Figure 11:**
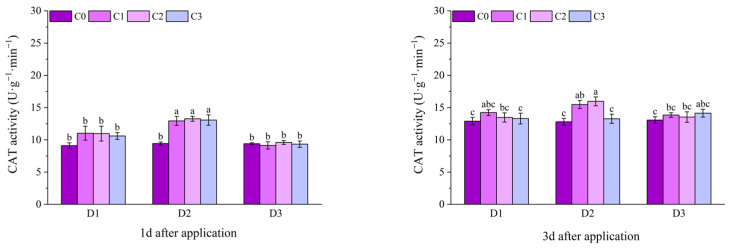

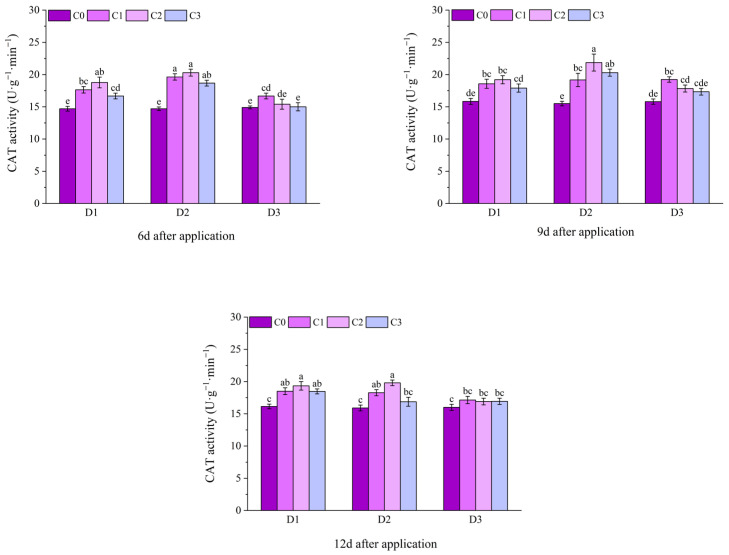
Effects of spraying BR on CAT activity of potted foxtail millet under cyhalofop-butyl damage. Comparison between treatments of different concentrations on the same day, with lowercase letters representing a significant difference (*p* < 0.05).

At 1 d after application, the CAT activities of all treatments with BR spraying under D2 of potted Jingu 21 were significantly higher than that of the D2C0 treatment, and there was no significant difference among other treatments. At 3 d after application, the CAT activities of the C1 and C2 treatments under D2 were significantly higher than that of the D2C0 treatment, the D2C2 treatment had the highest value, increasing by 24.74% compared with the D2C0 treatment, and there was no significant difference between other treatments and the C0 treatment. At 6 d after application, the CAT activities of all treatments with BR spraying under D1 and D2 were significantly higher than that of the C0 treatment. Compared with D1C0, the CAT activities of the treatments with BR spraying under D1 increased by 19.95%, 27.66% and 13.38%, respectively. Compared with D2C0, the CAT activities of the treatments with BR spraying under D2 increased by 33.56%, 38.10% and 26.98%, respectively. At 9 d and 12 d after application, the CAT activities of the C1 and C2 treatments under D1 and D2 were significantly higher than that of the C0 treatment. At 9 d after application, compared with D1C0, the CAT activities of the D1C1 and D1C2 treatments increased by 17.26% and 21.26%, respectively. Compared with D2C0, the CAT activities of the D2C1 and D2C2 treatments increased by 23.66% and 41.08%, respectively. At 12 d after application, compared with D1C0, the CAT activities of the D1C1 and D1C2 treatments increased by 14.70% and 19.83%, respectively. Compared with D2C0, the CAT activities of the D2C1 and D2C2 treatments increased by 14.88% and 24.53%, respectively (Figure 11).

**Figure 12:**
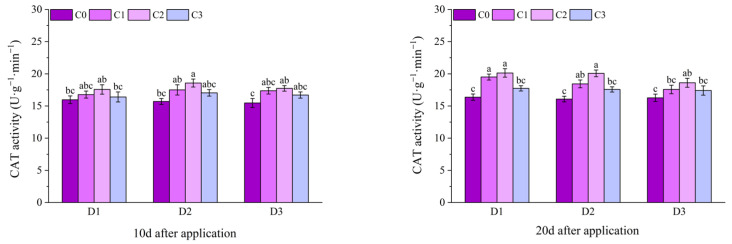

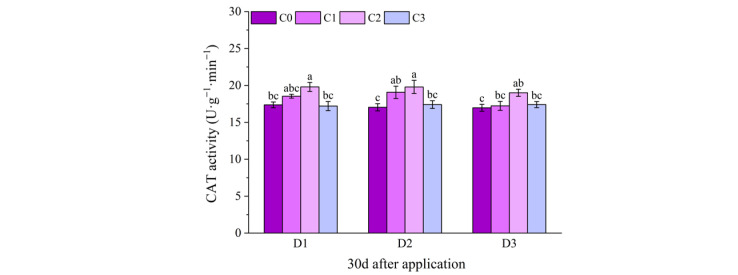
Effects of spraying BR on CAT activity of field foxtail millet under cyhalofop-butyl damage. Comparison between treatments of different concentrations on the same day, with lowercase letters representing a significant difference (*p* < 0.05).

At 10 d after application, the CAT activity of the D2C2 treatment of field Jingu 21 was the highest, increasing by 18.25% significantly compared with the D2C0 treatment. At 20 d and 30 d after application, the CAT activities of the C2 treatments under D1 and D3, and the C1 and C2 treatments under D2 were significantly different from that of the C0 treatment (Figure 12).

At 6 d after application, the spraying time of BR had an extremely significant effect on the SOD and CAT activities of potted Jingu 21. At 6 d, 9 d and 12 d after application, the spraying concentration of BR had an extremely significant effect on the SOD, POD and CAT activities of potted Jingu 21. At 12 d after application, the interaction between the spraying time and concentration of BR had no significant effect on the antioxidant enzyme activities (Table 3).

The spraying time of BR had no significant effect on the antioxidant enzyme activities of field Jingu 21. The spraying concentration of BR had an extremely significant effect on the SOD, POD, and CAT activities of Jingu 21 at 10 d and 20 d after application, as well as on the SOD and CAT activities at 30 d after application. The interaction between the spraying time and concentration of BR had no significant effect on the antioxidant enzyme activities of Jingu 21 (Table 4).

### 2.3. Effect of BR Spraying on MDA Content of Jingu 21 Under Cyhalofop-Butyl Damage

**Figure 13:**
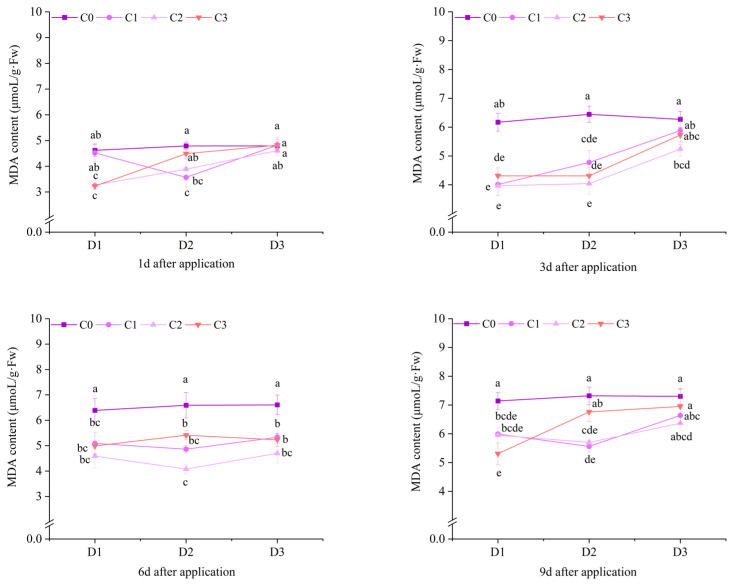

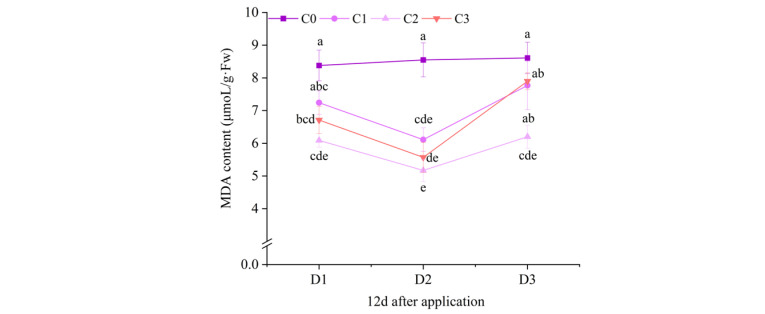
Effects of spraying BR on MDA contents of potted foxtail millet under cyhalofop-butyl damage. Comparison between treatments of different concentrations on the same day, with lowercase letters representing a significant difference (*p* < 0.05).

At 1 d after application, the MDA contents of the C2 treatments under D1 and D2 of potted Jingu 21 were significantly lower than that of the C0 treatment. At 3 d after application, the MDA contents of all treatments with BR spraying under D1 and D2 were significantly different from that of the C0 treatment. Compared with D1C0, the MDA contents of the treatments with BR spraying under D1 decreased by 35.05%, 35.74% and 30.15%, respectively. Compared with D2C0, the MDA contents of the treatments with BR spraying under D2 decreased by 25.91%, 37.36% and 33.13%, respectively. At 6 d, 9 d and 12 d after application, the MDA contents of all treatments with BR spraying under D1 and D2 were significantly lower than that of the C0 treatment. At 12 d after application, the MDA content of the D2C2 treatment reached the minimum value, decreasing by 39.56% compared with the D2C0 treatment (Figure 13).

**Figure 14:**
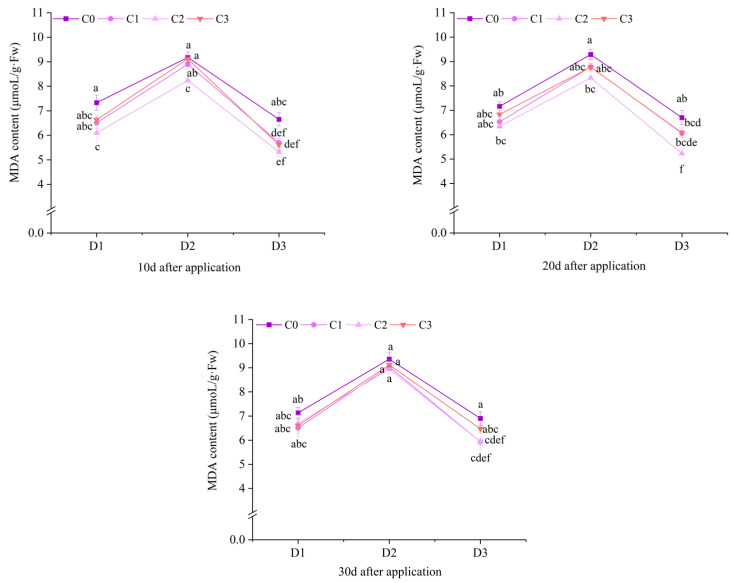
Effects of spraying BR on MDA contents of field foxtail millet under cyhalofop-butyl damage. Comparison between treatments of different concentrations on the same day, with lowercase letters representing a significant difference (*p* < 0.05).

At 10 d after application of field Jingu 21, except for the D1C2 treatment, there was no significant difference in MDA content between other treatments with BR spraying and the C0 treatment. At 20 d after application, the MDA contents of the C2 treatments under D1 and D2 were significantly different from that of the C0 treatment, decreasing by 10.22% and 10.29%, respectively, compared with the C0 treatment. At 30 d after application, the MDA contents of the treatments with BR spraying under D1, the D2C2 treatment, and the C1 and C2 treatments under D3 were significantly lower than that of the C0 treatment (Figure 14).

At 1 d, 3 d, 9 d and 12 d after application, the spraying time of BR had an extremely significant effect on the MDA content of potted Jingu 21. The spraying concentration of BR had an extremely significant effect on the MDA content of potted Jingu 21. Except for 1 d and 9 d after application, the interaction between spraying time and concentration of BR had no significant effect on the MDA content (Table 5).

The spraying time of BR had a significant effect on the MDA content of field Jingu 21 at 20 d and 30 d after application. The spraying concentration of BR had an extremely significant effect on the MDA content of Jingu 21 after application. The interaction between the spraying time and concentration of BR had no significant effect on the MDA content of Jingu 21 (Table 5).

### 2.4. Effect of BR Spraying on Drug Damage Level of Jingu 21 Under Cyhalofop-Butyl Damage

**Figure 15:**
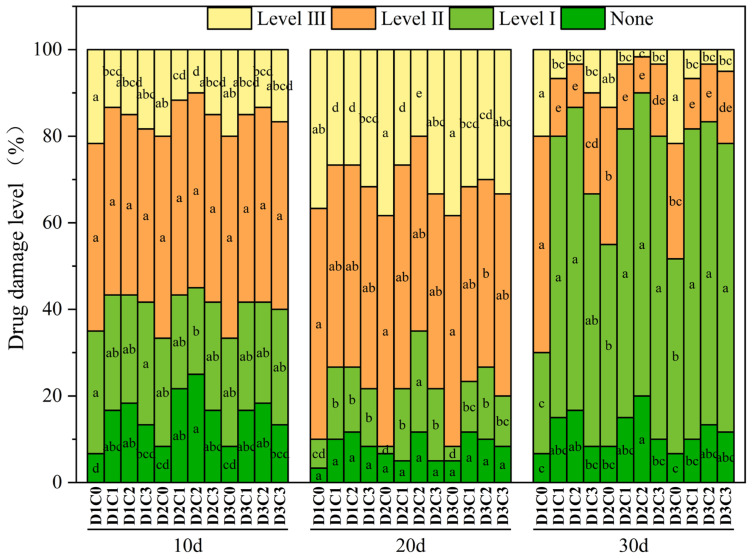
Effects of spraying BR on drug damage level of Jingu 21 under Cyhalofop-butyl damage. Comparison between treatments of different concentrations on the same day, with lowercase letters representing a significant difference (*p* < 0.05).

At 30 d after application, the alleviation degree of drug damage at level II and level III of field Jingu 21 was relatively large. At 30 d after application, compared with D1C0, the number of plants with drug damage level III in the treatments with BR spraying under D1 decreased by 66.67%, 83.33% and 50.00%, respectively, and the number of plants with drug damage level II decreased by 73.33%, 80.00% and 53.33%, respectively. Compared with D2C0, the number of plants with drug damage level III in the treatments with BR spraying under D2 decreased by 75.00%, 87.50% and 75.00%, respectively, and the number of plants with drug damage level II decreased by 52.63%, 73.68% and 47.37%, respectively. Compared with D3C0, the number of plants with drug damage level III in the treatments with BR spraying under D3 decreased by 69.23%, 84.62% and 76.92%, respectively, and the number of plants with drug damage level II decreased by 56.25%, 50.00% and 37.50%, respectively, compared with D2C0 (Figure 15).

### 2.5. Effect of BR Spraying on Yield of Jingu 21 Under Cyhalofop-Butyl Damage

**Figure 16:**
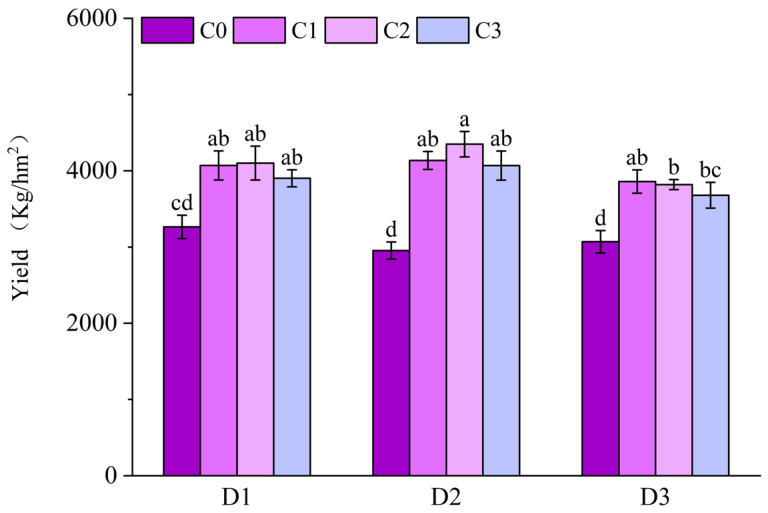
Effects of spraying BR on yield of Jingu 21 under Cyhalofop-butyl damage. Comparison between treatments of different concentrations on the same day, with lowercase letters representing a significant difference (*p* < 0.05).

After treatment with sprayed BR, the yield of Jingu 21 was increased to varying degrees. The yield was the highest under the D2C2 treatment, reaching 4349.98 Kg/hm^2^. The yields of treatments with BR spraying under D1 increased by 24.71%, 25.63% and 19.52%, respectively, compared to D1C0 treatment. The yields of treatments with BR spraying under D2 increased by 39.98%, 47.26% and 37.72%, respectively, compared to D2C0 treatment. The yields of treatments with BR spraying under D3 increased by 25.71% 24.42%, and 19.98%, respectively, compared to D3C0 treatment (Figure 16).

## 3. Discussion

Brassinolide is involved in regulating plant growth and development and plays an important role in plant cell elongation and division, leaf morphogenesis, and crop yield improvement [27]. Applying an appropriate concentration of BR can promote cell division and elongation and alleviate the inhibitory effect of herbicide stress on crop agronomic trait indicators [28]. Studies have found that, after BR spraying, the flower diameter and plant height of *Freesia hybrida* increased significantly [29]. Exogenous BR can regulate important agronomic traits [30,31,32] and significantly improve the seed germination rate, above-ground part and root length of seedlings, and fresh and dry weights [33,34]. Jingu 21 is a conventional variety that is mainly planted in Shanxi Province with poor herbicide resistance. Its plant height and leaf area, and the dry weight of aboveground parts, are seriously inhibited by damage. In this experiment, all agronomic trait indicators of Jingu 21 in both pot and field experiments were alleviated after BR spraying, indicating that BR spraying can improve the growth indicators of foxtail millet and cultivate strong seedlings. However, the responses to different BR concentration treatments are different. Spraying BR on the same day as cyhalofop-butyl spraying and one day after cyhalofop-butyl spraying had a better alleviating effect on the growth indicators of foxtail millet.

Reactive oxygen species (ROS) can damage cell membranes and other biological macromolecules. Antioxidant enzymes can scavenge a large amount of ROS released in foxtail millet due to stress, enhance the stress resistance of foxtail millet plants, and play an important role in maintaining the dynamic balance of ROS in plants. In addition, under stress conditions, the permeability of plant cell membranes will change accordingly, and the structure and function of cell membranes will be damaged to varying degrees [35,36]. MDA is one of the products of cell membrane lipid peroxidation and will increase significantly when plants are subjected to stress. Applying BR can enhance the ability of the crop antioxidant defense system and reduce membrane lipid peroxidation damage [23,37]. Studies have shown that spraying BR can increase SOD activity, promote the elongation of the mesocotyl, and increase the mesocotyl’s length and emergence rate, thereby improving deep-sowing tolerance [38]. After BR spraying, the SOD, POD, and CAT activities of *Freesia hybrida* increased [29]. BR treatment can significantly increase the SOD and CAT activities in cucumber leaves and effectively reduce the MDA content [16,39,40]. When subjected to herbicide stress, foxtail millet has a corresponding protection system for timely defense and response. The results of this experiment are similar to the previous research conclusions. After BR spraying, the SOD, POD, and CAT activities of foxtail millet increased to varying degrees compared with the treatment without BR spraying, and the MDA content decreased.

Brassinolide may activate brassinosteroid signal transduction and upregulate the expression of degradation genes, thereby increasing the activity of detoxifying enzymes [41], and promote the signal pathways involved in BRs-induced H_2_O_2_ production and cellular redox changes to facilitate pesticide metabolism, thus reducing pesticide residues in plants [42]. After being subjected to herbicide stress, spraying BR can promote the detoxification metabolism of crops, reduce the physiological damage of crops, and improve the degradation efficiency of crops on herbicides, which reduces the damage symptom grade of crops. Studies have shown that applying BR significantly increases the transcription of antioxidant, detoxification, and defense genes in tomatoes under chlorpyrifos damage, which can enhance the tolerance of tomatoes to pesticides and reduce toxicity [19]. The appropriate application of exogenous BR can effectively alleviate vanillin stress on *Lagenaria siceraria* and improve tolerance to vanillin stress [43]. The results of this experiment show that spraying BR can significantly reduce the proportion of Jingu 21 plants with drug damage levels II and III and increase the proportion of plants with drug damage level I, indicating that spraying BR has an alleviating effect on the damage of Jingu 21 plants and shortens the recovery period following cyhalofop-butyl damage in foxtail millet.

Brassinolide is a crucial class of plant growth regulators that can promote crop yield under stress conditions. They optimize the stress-resistant physiological traits of crops [17], alleviate the inhibitory effects of stress on crop growth, and reduce yield losses. Studies have shown that spraying brassinolide can significantly increase the grain yield and harvest index of hybrid rice [3]. BR treatment not only significantly improves the yield of *Pinellia ternata*, but also maintains a high level of total alkaloids in its tubers [14]. The results of this experiment indicate that spraying BR can significantly increase the yield of Jingu 21. Among all treatments, the application of 0.1 mg/L BR one day later resulted in the highest yield, and all other spraying BR treatments also achieved higher yields compared to the control group.

## 4. Materials and Methods

### 4.1. Materials

Test variety: Jingu 21 (a conventional variety not resistant to herbicides, bred by the Economic Crop Research Institute of Shanxi Academy of Agricultural Sciences)

Test agents: 100 g/L cyhalofop-butyl emulsifiable concentrate (Corteva Agricultural Science and Technology Co., Ltd., Langfang, China), 14-Hydroxylated brassinolide (active ingredient content: 0.01%) (Chengdu Xinzhaoyang Crop Science Co., Ltd., Chengdu, China)

Test soil: The test research base is located at the Crop Station of Shanxi Agricultural University (37°25′ N, 112°34′ E), with an altitude of 799.6 m. It belongs to a warm temperate continental climate. The soil used in the field experiment was sandy loam. The basic fertility of soil (Table 6) was determined by the Environmental Monitoring Center of Shanxi Agricultural University; the soil used in the pot experiment was Wode seedling substrate (executive standard: NY/T2115-2012).

### 4.2. Experimental Design

The same experimental treatments were set for Jingu 21 in both pot and field experiments (Table 7). The 3WP-2000 self-propelled spray tower (development of Agricultural Machinery Research Institute of Nanjing Academy of Agricultural Sciences, Nanjing, China) was used for pot experiment spraying. Agricultural electric spray was used for spraying in the field experiment. All test treatments were sprayed with cyhalofop-butyl at a concentration of 67.5 g a.i./ha. The water volume for diluting cyhalofop-butyl and brassinolide during spraying was 525 L/ha liters each. Three BR spraying times were set: the same day as cyhalofop-butyl spraying (D1), one day later (D2), and three days later (D3). Four BR concentrations were set: 0 mg/L (C0), 0.05 mg/L (C1), 0.1 mg/L (C2), and 0.2 mg/L (C3), resulting in a total of 12 treatments.

The pot experiment was conducted in the Crop Chemistry and Regulation Laboratory of Shanxi Agricultural University, and indicators were measured at 1 d, 3 d, 6 d, 9 d, and 12 d after cyhalofop-butyl spraying. The field experiment was conducted in the experimental field of the Agricultural Station of Shanxi Agricultural University, and indicators were measured at 10 d, 20 d, and 30 d after cyhalofop-butyl spraying.

### 4.3. Test Indicators and Analysis Methods

#### 4.3.1. Agronomic Traits

Foxtail millet plants with uniform growth were selected to measure the plant height (the height from the base of the foxtail millet plant to the flag leaf) and leaf area (leaf area = leaf length × leaf width × 0.75). A digital display electric blast drying oven was used to kill the samples at 105 °C for 30 min, and then the temperature was adjusted to 80 °C to dry the samples to a constant weight, and the above-ground dry weight of the plants was weighed.

#### 4.3.2. Antioxidant Enzyme Activities and MDA Content

A total of 0.1 g of the second leaf from the top of foxtail millet was weighed and crushed into powder, and 1.8 mL of phosphate buffer (pH 7.8) was added and mixed uniformly. The mixture was centrifuged in a low-temperature, high-speed centrifuge at 12,000 r/pm and 4 °C for 15 min, and the supernatant was taken for determination. Superoxide dismutase (SOD) activity was determined by the photochemical reduction method of nitroblue tetrazolium [44]. Peroxidase (POD) activity was determined by the guaiacol oxidation method [39]. Catalase (CAT) activity was determined by the hydrogen peroxide method [45]. The MDA content was determined by the thiobarbituric acid method [40]. A total of 0.2 g of the second leaf from the top of foxtail millet was weighed and ground into powder, and 2.5 mL of 0.1% trichloroacetic acid solution and 2.5 mL of 0.5% thiobarbituric acid solution were added in sequence. The mixture was shaken well, heated in a boiling water bath for 15 min, and then immediately taken out and cooled to room temperature in ice water. After centrifugation at 3000 r/pm for 15 min, the supernatant was taken out, its volume was measured, and the absorbance values were measured at 532 nm and 600 nm, respectively.

#### 4.3.3. Drug Damage Level

Thirty foxtail millet plants were randomly selected in each test plot to determine the drug damage level. The damage grade standards are as follows:

None: The plants grow normally, the leaves are healthy, and there is no obvious damage.
Level I: Less than 20% of the leaves of foxtail millet are withered and yellow or have spots.
Level II: 20–50% of the leaves of foxtail millet are withered, yellow, and curled.
Level III: 50–70% of the leaves of foxtail millet are withered, yellow, and deformed.
Level IV: More than 70% of the leaves of foxtail millet are withered or the plants die.

#### 4.3.4. Yield

Samples of foxtail millet were collected from each treatment group. The sampling area for each sample was 4 m^2^, with 3 replicates per treatment. The foxtail millet grains were accurately weighed subsequent to threshing, and the yield per hectare was further derived.

### 4.4. Data Processing

Microsoft Excel 2021 (Microsoft, Redmond, WA, USA) and IBM SPSS Statistics 25 (SPSS Inc., Chicago, IL, USA) software were used for data processing. Duncan’s new multiple range test was used to test the significance level of differences among treatments and conduct variance analysis (*p* < 0.05). Origin 2021 (OriginLab, Northampton, MA, USA) software was used to draw experimental charts to ensure the intuitiveness and accuracy of data presentation. The letters marking significant differences in all figures and tables in this paper are comparisons among 12 treatments, with 4 repetitions for each treatment.

## 5. Conclusions

Different BR spraying times and BR concentrations had a certain alleviating effect on the plant height, leaf area, and dry weight of aboveground parts of Jingu 21. The BR concentration had an extremely significant effect on the agronomic traits of foxtail millet. With the increase in BR concentration, the alleviating effect on the agronomic trait indicators of Jingu 21 showed a trend of first increasing and then decreasing. BR at a concentration of 0.1 mg/L could significantly increase the antioxidant enzyme activity of Jingu 21, reduce the MDA content, and significantly reduce the proportion of Jingu 21 plants with drug damage level II and level III, indicating that BR spraying had an alleviating effect on the damage of Jingu 21 plants. In addition, spraying BR can increase the yield of Jingu 21 under cyhalofop-butyl herbicide damage.

In conclusion, spraying BR at a concentration of 0.1 mg/L can effectively alleviate the cyhalofop-butyl damage of Jingu 21. Spraying BR one day after cyhalofop-butyl spraying had the best effect, followed by spraying BR on the same day as cyhalofop-butyl spraying, and spraying BR three days after cyhalofop-butyl spraying had the worst effect. It is recommended that when using BR to alleviate the damage of foxtail millet, the application should be spaced one day apart from the herbicide spraying.

## Figures and Tables

**Table 1:** Variance analysis of agronomic traits of potted foxtail millet under cyhalofop-butyl damage by spraying BR.

Analysis of Variance		Plant Height	Leaf Area	Dry Weight of Aboveground Parts
spraying time (D)	1 d	NS	NS	NS
	3 d	**	NS	NS
	6 d	**	NS	*
	9 d	**	NS	*
	12 d	*	**	**
spraying concentration (C)	1 d	**	NS	NS
	3 d	**	NS	**
	6 d	**	NS	**
	9 d	**	**	**
	12 d	**	**	**
Time × concentration (D × C)	1 d	NS	NS	NS
	3 d	*	NS	NS
	6 d	NS	NS	NS
	9 d	NS	NS	NS
	12 d	NS	NS	**

Note: The “*” and “**” respectively indicate significant and extremely significant differences at the 0.05 and 0.01 levels, while “NS” indicates no significant difference.

**Table 2:** Variance analysis of agronomic traits of field foxtail millet under cyhalofop-butyl damage by spraying BR.

Analysis of Variance		Plant Height	Leaf Area	Dry Weight of Aboveground Parts
spraying time (D)	10 d	*	**	NS
	20 d	NS	*	**
	30 d	NS	NS	**
spraying concentration (C)	10 d	**	**	**
	20 d	**	**	**
	30 d	**	**	**
Time × concentration (D × C)	10 d	**	**	NS
	20 d	NS	NS	NS
	30 d	NS	NS	**

Note: The “*” and “**” respectively indicate significant and extremely significant differences at the 0.05 and 0.01 levels, while “NS” indicates no significant difference.

**Table 3:** Variance analysis of antioxidant enzymes of potted foxtail millet under cyhalofop-butyl damage after spraying BR.

Analysis of Variance		SOD Activity	POD Activity	CAT Activity
spraying time (D)	1 d	**	**	**
	3 d	**	NS	NS
	6 d	**	NS	**
	9 d	NS	NS	**
	12 d	NS	NS	**
spraying concentration (C)	1 d	**	*	**
	3 d	**	NS	*
	6 d	**	**	**
	9 d	**	*	**
	12 d	**	**	**
Time × concentration (D × C)	1 d	*	*	NS
	3 d	**	NS	NS
	6 d	NS	NS	**
	9 d	NS	NS	*
	12 d	NS	NS	NS

Note: The “*” and “**”indicate significant and extremely significant differences at the 0.05 and 0.01 levels, respectively, while “NS” indicates no significant difference.

**Table 4:** Variance analysis of antioxidant enzymes of field foxtail millet under cyhalofop-butyl damage after spraying BR.

Analysis of Variance		SOD Activity	POD Activity	CAT Activity
spraying time (D)	10 d	NS	NS	NS
	20 d	NS	NS	NS
	30 d	NS	NS	NS
spraying concentration (C)	10 d	**	**	**
	20 d	**	**	**
	30 d	**	NS	**
Time × concentration (D × C)	10 d	NS	NS	NS
	20 d	NS	NS	NS
	30 d	NS	NS	NS

Note: The “**” indicate extremely significant differences at the 0.01 levels, while “NS” indicates no significant difference.

**Table 5:** Variance analysis of MDA content of foxtail millet under cyhalofop-butyl damage by spraying BR.

Analysis of Variance		Spraying Time (D)	Spraying Concentration (C)	Time × Concentration (D × C)
pot experiments	1 d	**	**	**
	3 d	**	**	NS
	6 d	NS	**	NS
	9 d	**	**	*
	12 d	**	**	NS
field experiments	10 d	NS	**	NS
	20 d	*	**	NS
	30 d	*	**	NS

Note: The “*” and “**” respectively indicate significant and extremely significant differences at the 0.05 and 0.01 levels, while “NS” indicates no significant difference.

**Table 6:** Basic fertility of soil tested.

Years	pH	Available P mg/kg	Available K mg/kg	Total N g/kg	Total P g/kg	Total K g/kg	Organic Matter g/kg	Hydrolyzable Nitrogen mg/kg
2024	8.29	13.4	189.98	1.01	0.81	20.44	17.26	69.74

**Table 7:** Setting scheme of experiment treatment.

Spraying Amount of Cyhalofop-Butyl	Time of Spraying BR	Concentration of Spraying BR
67.5 g a.i./ha	The same day as cyhalofop-butyl spraying (D1)	0 mg/L (C0)
		0.05 mg/L (C1)
		0.1 mg/L (C2)
		0.2 mg/L (C3)
	One day after cyhalofop-butyl spraying (D2)	0 mg/L (C0)
		0.05 mg/L (C1)
		0.1 mg/L (C2)
		0.2 mg/L (C3)
	Three days after cyhalofop-butyl spraying (D3)	0 mg/L (C0)
		0.05 mg/L (C1)
		0.1 mg/L (C2)
		0.2 mg/L (C3)

## Data Availability

The data that support this study are available upon reasonable request from the corresponding authors.
